# Sustainability and Localization of Health Infrastructure under CPEC 2.0: Toward a Self-Reliant Health System in Pakistan

**DOI:** 10.12669/pjms.42.5.16529

**Published:** 2026-05

**Authors:** Yang Rui, Qu Qiumei

**Affiliations:** 1 Yang Rui Lecturer, School of Maxism, Yunnan Technology and Business University, Kunming, China; 2 Qu Qiumei Visiting Lecturer, Department of History & Pakistan Studies, University of the Punjab, Lahore, Pakistan

**Keywords:** CPEC2.0, Healthcare Infrastructure, Sustainability, Localization, Project-driven, System Embedding

## Abstract

The Economic Corridor is being developed as part of a strategic partnership between the governments of China and Pakistan, which was announced during the visit of Prime Minister of Pakistan to China in July, 2013. China-Pakistan Economic Corridor is a fusion of multiple corridors of Investment, Trade, Energy, Transport, Infrastructure and Industrial Cooperation. The first phase of the China-Pakistan Economic Corridor (CPEC) focused primarily on investment, trade, energy, transportation, infrastructure, and industrial cooperation. CPEC 2.0 emphasizes sectors such as agriculture, electric vehicles, solar energy, healthcare, and steel. Incorporating healthcare infrastructure into CPEC 2.0 represents not merely an expansion of cooperative scope but a structural evolution from “project-driven” to “system-embedded” healthcare infrastructure development. This article analyzes the three-dimensional sustainability model and four-aspect localization framework. Findings indicate that CPEC 2.0 provides a strategic platform for Pakistan to develop indigenous capabilities in critical areas such as financing, regulation, talent cultivation, equipment maintenance, and technological innovation. However, its long-term effectiveness hinges on the resilience of local institutions, policy continuity, and the overall stability of the economic structure.

On November 13, 2016, the first convoy from China arrived at Gwadar Port, marking the official launch of the China-Pakistan Economic Corridor.^1^ The China-Pakistan Economic Corridor is regarded as a core pillar of China’s Belt and Road Initiative.^2^ By early 2024, it had become the most developed land corridor within the Belt and Road Initiative.^3^ Recent events therefore point to a renewed sense of urgency and strategic focus on pushing CPEC Phase 2.0, the next phase of the project. Through increased cooperation, cutting-edge technology transfer, and revolutionary socioeconomic initiatives that anticipate long-term prosperity for both countries, this crucial stage seeks to alter the framework of bilateral ties.

In September 2025, during Pakistani Prime Minister Shehbaz Sharif’s visit to Beijing, China and Pakistan signed agreements worth approximately $8.5 billion covering agriculture, electric vehicles, solar energy, healthcare, and steel.^4^ Both countries continue working together on livelihood-related projects in fields like education, health, agriculture, disaster prevention and mitigation, and climate response. To increase the two peoples’ perception of the benefits of China-Pakistan cooperation, more “small and beautiful” livelihood initiatives will be carried out throughout Pakistan.^5^

Medical infrastructure has become a key component of CPEC 2.0, shifting from “project-driven” to “system-embedded.” Its sustainability and localization represent not merely sectoral adjustments, but a structural reconfiguration of the corridor’s developmental logic. This article analyzes its sustainability across four dimensions: institutional, financial, technical, and human resources.

This study focuses on the localization and sustainability of healthcare infrastructure within the context of the China-Pakistan Economic Corridor (CPEC). It offers practical solutions for the structural evolution of healthcare infrastructure development in CPEC 2.0-moving from “project-driven” to “system-embedded” approaches-across three sustainability dimensions and four localization dimensions by fusing qualitative literature analysis with comparative policy evaluation.

Official policy documents from Chinese government agencies, including the State Council, National Development and Reform Commission, Ministry of Foreign Affairs, and National Health Commission, were among the many trustworthy sources of data and materials used in this study. The World Health Organization (WHO), Pakistan’s Ministry of Planning, the Economic and Commercial Office of the Chinese Embassy in the Islamic Republic of Pakistan, and the National Agency for International Development Cooperation are among the international organizations whose reports and guidelines are included in the scope. Institutions like Jianpei Technology and the China Association for Science and Technology contributed supplementary academic research and policy writing. Today Pakistan, China Daily, Xinhua News Agency, and China Economic Net are examples of pertinent media sources.

The authors carefully considered the materials’ publication dates, credibility, and appropriateness for the localization and sustainability of medical infrastructure development in Phase 2.0 of the China-Pakistan Economic Corridor while choosing them.

## Financial Sustainability:

A population’s standard of living is driven by both social and economic well-being. By emphasizing the social and economic welfare of Pakistanis, the corridor of livelihood seeks to establish a society founded on the normative values of empowerment, diversity, equality, and inclusion.

CPEC 2.0 designates digital medical and healthcare as one of the key project directions for the “People’s Livelihood Corridor,” integrating public services (including healthcare) into the long-term investment framework. This necessitates the reallocation of public finances to support the sustainable operation of these “soft infrastructure” projects. Fiscal arrangements must not only fund one-time construction but also sustain long-term operation and maintenance.

Challenges in sustainable financing—including weak tax mobilization capacity, insufficient prioritization of the health sector, and significant funding gaps in expanding the provision of essential health service packages. According to the most recent statistics available in 2021, Pakistan’s service coverage index (SCI) score nearly doubled from 22 in 2000 to 45 in 2021, although it is still much lower than the average score of 58 among lower middle-income nations.^6^

It is evident that the fiscal sustainability of healthcare infrastructure is structurally constrained by two factors: weak tax mobilization capacity and insufficient prioritization of the health sector. Limited fiscal budgets have compelled the Pakistani government to refrain from consistently prioritizing healthcare infrastructure. However, the structure of fiscal priorities determines the long-term sustainable operation of healthcare infrastructure. Therefore, CPEC 2.0 must prioritize increasing sustainable funding for healthcare infrastructure during cooperation efforts.

With an emphasis on domestic pharmaceutical manufacturing, Traditional Chinese Medicine (TCM), and vital vaccine supply, Pakistan has promised to fully facilitate Chinese investments in the healthcare industry. “We are prepared to provide Chinese partners in the healthcare industry with full support and facilitation,”^7^ said Kamal. The minister declared that Pakistan’s National Institute of Health (NIH) will set up a special China desk in order to formalize this pledge. The investment frameworks, regulatory alignments, and deadlines for deliverables under the new project are finalized by officials from both nations.

This collaboration demonstrates that China-Pakistan health cooperation is transitioning from political commitments to institutional and financial coordination. By establishing specialized liaison mechanisms like TCM and defining clear investment frameworks, this cooperative model helps alleviate pressure on Pakistan’s public finances in the health sector.

## Institutional Sustainability:

The Prime Minister of Pakistan emphasized that newly constructed hospitals must prioritize high-quality medical facilities to ensure the provision of sustainable, patient-centered healthcare services following the Chinese model. China has exerted a direct influence on Pakistan’s healthcare governance and institutional development.^8^ The China-Pakistan Friendship Hospital is a significant people-oriented assistance project under the China-Pakistan Economic Corridor framework and a major cooperative achievement in the high-quality joint construction of the Belt and Road Initiative. As a new-standard medical institution, its operations must comply with Pakistani healthcare service regulatory requirements. In practice, it may also drive local health authorities to strengthen their institutional enforcement and oversight capabilities. China’s management standards, service protocols, and quality control requirements for foreign-funded hospitals will exert practical pressure on the implementation of relevant Pakistani regulations, thereby contributing to greater standardization within the local healthcare sector.^8^ As Pakistan’s key health regulatory body, the NIH has established a dedicated task force for Chinese enterprises. This initiative is expected to enhance institutional transparency and procedural standardization for Chinese companies entering Pakistan’s healthcare market, while facilitating the development of relevant approval processes, standards, and regulatory mechanisms on the Pakistani side. This mechanism embodies the establishment of communication and cooperation between the two parties at the institutional level.

## Technological Sustainability:

Pakistan National Digital Health Framework 2022-2030^9^ Clearly positioning digital technology as the core strategic pathway for achieving sustainable development in healthcare systems. This framework proposes building a nationally interoperable digital health ecosystem. Through standardized data governance and information sharing mechanisms, it aims to enhance the operational efficiency and long-term resilience of healthcare service systems. Regarding the concrete implementation of technological sustainability, the framework particularly emphasizes improving digital health literacy and technological capacity building. This includes enhancing the understanding, usage, and management capabilities of digital technologies among healthcare professionals at all levels and the general public; establishing education and training mechanisms to transform technology from an external dependency into an endogenous growth driver that can be sustainably operated locally. Simultaneously, enhancing policy understanding and public awareness of digital technologies promotes broader societal acceptance and utilization rates, thereby reducing institutional friction and efficiency losses following technology adoption. Furthermore, the framework underscores leveraging digital technologies to optimize healthcare resource allocation. This includes extending service coverage to underserved areas through telemedicine, digital monitoring platforms, and information systems. Such measures not only elevate the quality of national health data but also strengthen the evidence base for health planning and performance evaluation, providing long-term, sustainable technological support for fiscal and operational decision-making.

## Local Talent Development:

For health systems to operate effectively, there must be a sufficient number of high-quality, productive healthcare professionals. In the region and the world, Pakistan has one of the lowest concentrations and numbers of vital health workers. By 2030, Pakistan aims to have 942,511 nurses, LHVs, and midwives and 314,170 physicians.^10^

Funding and talent are the two fundamental pillars for building the “Health Silk Road.” The underdeveloped economic conditions in less-developed regions along the Belt and Road constrain investment in construction funds. Whether it’s improving basic healthcare standards or enhancing emergency medical management capabilities, stable and sustained diversified funding is urgently needed. Simultaneously, there exists a significant shortage of multidisciplinary medical professionals in Belt and Road countries. Take the China-Pakistan Medical Emergency Center in Gwadar Port as an example: the center requires not only specialized medical personnel but also public health policy experts and Red Cross liaison officers. Due to underdeveloped economies, some Belt and Road countries face poor educational and healthcare environments, making it difficult to cultivate local talent or attract foreign professionals—a situation that hinders the advancement of medical standards.

Therefore, it is essential to strengthen local talent development by leveraging Pakistan’s needs and the resources and expertise of relevant medical organizations and academic institutions. This should involve cultivating professionals in preventive medicine, health inspection, and quarantine through diverse methods and channels to enhance the resilience of the China-Pakistan infectious disease prevention and control system. The response to COVID-19 demonstrates that a nation’s capacity to address major sudden biological security risks depends on its comprehensive strength, not merely on infectious disease prevention technologies themselves. Cooperation between the two countries in this field requires further reinforcement.

Attention must also be paid to providing corresponding technical skills training for relevant Pakistani personnel. China can dispatch outstanding domestic medical technicians to Pakistan for guidance, or establish technical guidance training programs to conduct specialized technical training, thereby enhancing the professional capabilities of technical personnel in the recipient country. During training, cultural differences, language barriers, and variations in learning habits between the two countries must be carefully considered. Training content should be tailored to the actual skill levels and genuine needs of Pakistani trainees to stimulate their interest and maximize training effectiveness. Additionally, both sides can collaborate with domestic hospitals and universities through foreign aid projects to offer training courses in China, providing Pakistani health technicians with opportunities and platforms for study in China and attracting them to pursue further education here.

## Equipment Maintenance Capability:

The constraints of hard infrastructure resources pose a significant challenge to China’s “Health Silk Road” initiative. Underdeveloped regions along the Belt and Road generally suffer from lagging infrastructure, including underdeveloped transportation networks, low transport efficiency, inadequate communication facilities, and low telephone and internet penetration rates. Some countries also face insufficient power supply, hindering the development of healthcare systems. Take Pakistan as an example: road passenger and freight transport account for 90% and 96% of total transportation volume, respectively. However, its road density is only 0.32 kilometers per square kilometer, far below that of South Asian neighbors like India, Bangladesh, and Sri Lanka. This significantly increases the difficulty of transporting medical supplies. Additionally, Pakistan’s aging power grid suffers from a high comprehensive line loss rate of 20% in transmission and distribution, leading to tight electricity supply and frequent large-scale blackouts. These power shortages and supply instability severely disrupt the operation of medical equipment and the normal conduct of medical work.

It is recommended that the scope of bilateral cooperation extend beyond healthcare and health-related scientific and technological fields to encompass the development of “infrastructure” that supports holistic physical and mental well-being throughout the entire life cycle. Scientific and technological cooperation, alongside economic and trade cooperation, form the two vital pillars of building the “Health Silk Road”: Science and technology represent the primary productive force, and scientific cooperation serves as the capacity guarantee for “health” development, determining the height and depth of the “Health Silk Road.” The economy provides the solid foundation for the coordinated advancement of material and spiritual civilization, while economic and trade cooperation act as the strength guarantee for ‘health’ development, determining the breadth and scope of the “Health Silk Road.”

## Localization of Management Authority:

The Chinese government must give full consideration to the issue of management authority during the construction of its overseas medical assistance network. An effective management mechanism ensures the smooth operation of resources such as capital, technology, and personnel. Among these, personnel management should be the top priority. Taking the aforementioned emergency center in Gwadar Port as an example: Currently, Chinese medical aid personnel at the Gwadar Port Emergency Center rotate every six months, making it difficult to ensure the standardization and continuity of management systems. How to fully motivate medical team members to demonstrate initiative has become the primary challenge Pakistan faces in personnel management.

## Domestic Substitution in Supply Chains:

The Geneva conference is part of a broader trend of enhanced cooperation between Islamabad and Beijing under the China-Pakistan Economic Corridor (CPEC) framework, which has now incorporated the Health Corridor into its new priority areas. This collaboration comes as Pakistan seeks to diversify its healthcare investments, reduce reliance on Western pharmaceutical imports, and bolster domestic production amid persistent supply shocks and rising healthcare costs.^7^ The following diagram clearly illustrates the logical relationship between them:

The reason for Pakistan’s low spending is that the government only devotes a small portion of its overall budget to health care, which is insufficient to provide everyone with access to basic, high-quality healthcare. In 2020-21,^11^ Pakistan’s public health spending (Rs 656 billion, or $4.1 billion) accounted for around 6% of all government spending, compared to an average of 10% in developing nations and 15% in high-income countries.^12^

In Pakistan, out-of-pocket expenses account for 51.9 percent of overall health expenditures,^13^ compared to the global average of roughly 15 percent, due to low levels of government spending. In addition to pushing some people into poverty or trapping them there, these payments discourage others from seeking necessary health care.

**Figure:**
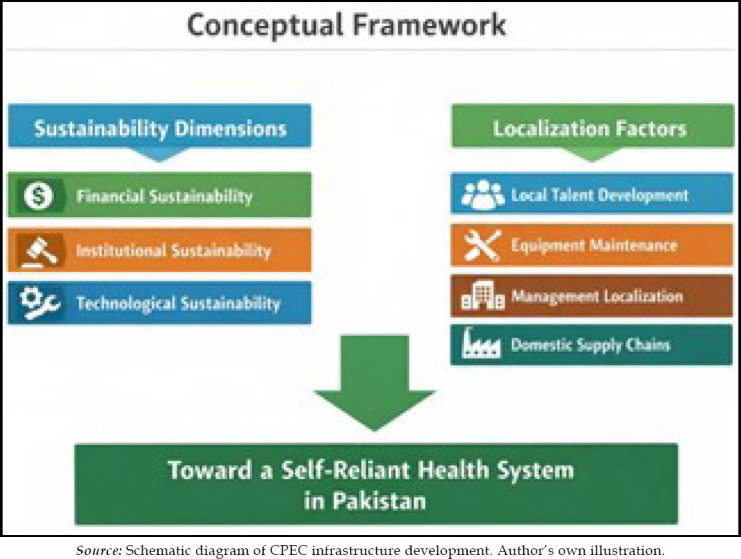
Source: Schematic diagram of CPEC infrastructure development. Author’s own illustration.

Pakistan would need a robust combination of foreign and domestic investment to close the significant UHC finance deficit. In its efforts to assist Pakistan in developing its medical network, China’s current funding primarily comes from the Silk Road Humanitarian Fund established by the Red Cross Society of China. The primary purpose of establishing this fund is to provide medical-related humanitarian services to countries along the Belt and Road, such as setting up first-aid stations and training medical personnel. The fund itself is primarily financed through donations and contributions from domestic and international individuals, legal entities, and other organizations. However, establishing an effective emergency medical network spanning thousands of miles requires additional funding sources.

## Human Resource Localization:

Any significant improvement in the health of Pakistanis depends on Human Resources for Health (HRH). A motivated and well-trained health workforce is essential to reaching the Sustainable Development Goals (SDGs) 2030 agenda and serves as the foundation for Universal Health Coverage (UHC). The foundation of Pakistan’s health systems and economic expansion is the country’s medical staff. Healthy societies are ensured by well-functioning health systems, which subsequently serve as a catalyst for economic growth.^13^ Possessing highly qualified medical personnel with strong technical expertise is crucial for enhancing the service capacity and improving the quality of the medical network. Each medical site requires specific personnel. Taking the emergency center in Gwadar Port, Pakistan, as an example, the medical team must include not only specialized healthcare providers but also public health policy officers and Red Cross liaisons. In short, Pakistan requires a well-trained, community-based, multi-skilled health workforce along with the corresponding support systems. This will also ensure that essential health services are delivered right at the doorstep of community members.

## Technological Maintenance and Supply Chains:

Staff from various disciplines—technical, clinical, financial, administrative, etc.—must be involved in medical device management. All employees who work with healthcare technology have an obligation to do this, not just supervisors.

It takes time to build the human resources required to run a successful maintenance program. The first step is to determine how many and what kind of employees are needed in a facility or collection of facilities. In this instance, facility level is equally crucial. For instance, a tiny medical facility with simpler technology and a comparatively little inventory can function with just one technician (as in the case of related facilities which HMC). However, a sizable tertiary care or specialty hospital will require a sophisticated clinical engineering department with a large number of technical and managerial staff, including experts in specific technologies, and numerous layers of oversight (as in the case of AKU). However, there are often two types of clinical engineering staff: managerial and technical. Finding the right mix of engineers and technicians is also influenced by the organization’s financial capability and the talents available in the local market. External service providers (either the vendor/manufacturer’s service representatives or third-party service representatives) must be added to the internal workforce in almost all maintenance plans. Under the direction of the employees of the in-house biomedical department, the service providers offer the IPM and CM for both highly advanced equipment that needs specialized training and equipment that is still under warranty.

## Institutional Integration:

The National Health Strategy (2023-2030) launched by the new government paints an ambitious blueprint: a $5 billion investment to upgrade medical infrastructure, with a focus on building regional treatment centers and telemedicine networks. However, funding mechanisms remain unclear, with confirmed international aid currently accounting for only 18% of the total budget. Land acquisition challenges also hinder project implementation—a planned oncology hospital near Islamabad has faced a two-year delay due to opposition from religious groups.

Public-private partnerships (PPPs) are seen as key to breaking the deadlock, but implementation outcomes have been mixed. In early pilot projects, Punjab’s Cardiovascular Disease Prevention and Treatment Center achieved success by adopting Singaporean management models, boosting bed turnover rates by 40%. Conversely, Sindh’s Mental Health Complex incurred losses due to the contractor’s lack of experience. This stark contrast serves as a warning that institutional design must fully account for localization and adaptation.^14^

## Challenges to Self-Reliance:

Pakistan’s health care system has not been able to function to its full potential despite ongoing efforts to build a vast primary health care network. This is primarily due to a lack of human resources, poor management, low budget, insufficient spending, and an unfair distribution of resources between the country’s urban and rural areas.^15^

Pakistan is among the developing nations whose healthcare systems are barely scraping by. Pakistan is regrettably still lagging behind the many other South Asian nations that have joined the global community in the fight against global health challenges. In this state, the most neglected sectors are health and education. The Human Development Report from 2006 states that 50% of individuals are literate, with 53% of those over the age of ten being literate.^15^ Pakistan’s economy and health sector have showed an increasing but slow trend over the past 20 years, but the country is unable to meet the population’s health demands because of ongoing political, terrorist, and corrupt issues. Additionally, the health sector struggles to generate people and necessary supplies due to limited resources and improper financial allocation. Additionally, economic development is a major contributing element to both international migration and brain drain.

Although the definition of primary health care in Pakistan makes it clear that all citizens should have universal access to care through socially accessible methods and technologies, this idea may be too far from reality. With the advent of science, technology, and continuing medical research, the impact has increased both locally and globally, increasing people’s quality of life. Biomedical innovations including telemedicine, e-health systems, bioinformatics, and biomedical imaging are used for diagnosis and treatment in the majority of developing nations. Pakistan lags far behind in terms of technological development. The nation urgently needs to create a health information technology environment nationwide and undergo a transition in health care.

Against the backdrop of the China-Pakistan Economic Corridor (CPEC) entering its 2.0 phase, healthcare infrastructure cooperation is shifting from “project-driven construction” to “institutionally embedded development.” This analysis examines three dimensions of sustainability—fiscal, institutional, and technological—alongside four dimensions of localization: local talent development, equipment maintenance capabilities, management rights localization, and domestic substitution in the supply chain, culminating in a comprehensive assessment.

First, regarding fiscal sustainability, transitioning from political commitment to institutional fiscal coordination will help alleviate pressure on Pakistan’s public finances in the health sector. Simultaneously, diversifying revenue sources and integrating expanded financial sustainability into the regular budget system will enhance the efficiency and equity of healthcare access while strengthening financial safeguards.

Second, regarding institutional sustainability, Pakistan’s establishment of a dedicated task force for Chinese enterprises is expected to enhance institutional transparency and procedural standardization for Chinese companies entering Pakistan’s healthcare market. This will facilitate the development of relevant approval processes, standards, and regulatory mechanisms by the Pakistani side. This mechanism embodies the establishment of communication and cooperation between the two sides at the institutional level.

Finally, regarding human resources and skills sustainability, Pakistan explicitly views digital technology as a crucial pillar for achieving national health system sustainability. Through multi-tiered measures—including establishing interoperable data platforms, promoting intelligent inventory management systems and advanced analytical tools, and strengthening command and communication systems—the country aims to enhance the efficiency of healthcare resource utilization and system resilience. Simultaneously, the framework advocates for the gradual integration of artificial intelligence, big data, and Internet of Things technologies, proposing data-driven decision-making and multi-stakeholder collaborative governance as institutional safeguards for sustainability. This strategy underscores Pakistan’s transition toward a self-reliant and high-performing health system.

In summary, the CPEC 2.0 phase marks a strategic upgrade in China-Pakistan healthcare cooperation, shifting from “infrastructure export” to “institutional co-construction and capacity transfer.” CPEC 2.0 provides a strategic platform for Pakistan’s transformation toward building endogenous capabilities in critical areas such as financing, regulation, talent development, equipment maintenance, and technological innovation. However, its long-term effectiveness hinges on the resilience of local institutions, policy continuity, and the overall stability of the economic structure.
